# Safety of maternal creatine supplementation: evidence from a guinea pig model of full-term pregnancy

**DOI:** 10.1080/15502783.2025.2533674

**Published:** 2025-07-20

**Authors:** Alice K. Freeman, Rebecca M. Dyson, Mary J. Berry, Stacey J. Ellery

**Affiliations:** a University of Otago, Department of Paediatrics and Child Health, Wellington, New Zealand; b University of Otago, Department of Anatomy, Dunedin, New Zealand; c The Ritchie Centre, Hudson Institute of Medical Research, Clayton, Victoria, Australia; d Monash University, Department of Obstetrics and Gynaecology, Clayton, Victoria, Australia

**Keywords:** Maternal nutritional physiological phenomena, pregnancy, fetal growth, creatine

## Abstract

**Background:**

Maternal creatine supplementation, whereby a mother consumes creatine throughout pregnancy to increase fetal creatine levels, has been proposed as a prophylactic therapy for improving developmental outcomes in cases of fetal growth restriction, preterm birth, or perinatal hypoxia. However, data on the safety of creatine supplementation during normal pregnancy and its impact on term-born offspring are limited. This study evaluated the safety of maternal creatine supplementation using pregnant guinea pigs, a well-established translational model due to its precocial development.

**Methods:**

From day 21 of gestation (term ~69 days), pregnant Dunkin Hartley guinea pigs were orally supplemented with 0.3g/kg/day creatine monohydrate (n=27), or the equivalent volume of water (n=29). Fetal growth was tracked via repeat ultrasound, before offspring were either collected via caesarean section on gestational day (GD) 63 or delivered spontaneously at term. Term-born offspring were allocated to neonatal necropsy or juvenile cohorts, with those in the juvenile group undergoing an oral glucose tolerance test and DXA body composition analysis before sacrifice on postnatal day (PND) 28. Data were analysed by two-way ANOVA, Fisher’s exact test, two-tailed student t-test or area under the curve analysis, with significance considered at p<0.05.

**Results:**

Maternal creatine supplementation had no significant impact on gestational weight gain, fetal growth (biparietal diameter or abdominal circumference), or blood flow (umbilical or middle cerebral artery doppler). Offspring birthweights did not differ with supplementation at GD63 or at term, and no effect was seen on pregnancy length or stillbirth rate. Metabolically, creatine supplementation did not impact offspring glucose tolerance at PND21, or DXA body composition at PND28.

**Conclusion:**

These data support maternal creatine supplementation as being safe during healthy, term pregnancies and provide a foundation for its potential use in human pregnancy.

